# Supplementary material for: Facebook addiction and affected academic performance among Ethiopian university students: A cross-sectional study
Source: PLoS One. 2023 Feb 6;18(2):e0280306. doi: 10.1371/journal.pone.0280306 (PMC9901753; doi:10.1371/journal.pone.0280306)
Supplement: Attachment — Submitted filename: please see the format of writing for the journal.docx [file pone.0280306.s002.docx]

01.06.2022

To the Editor

PLOS ONE

Dear Editor,

Thank you very much for inviting me to read the paper titled, "Facebook addiction and affected academic performance among Ethiopian university students: A cross-sectional Study". It is of course a very important area of study and I think the study is a timely one considering contemporary social changes due to technological progressions.

Overall, as a reader of the paper, I felt that the paper **is written critically enough h**owever, there are some issues that I felt is needed to be modified in the current paper before publishing. I have added my detail comments on a separate paper labeled as “**Sheet for Comments**”.

**Congratulations to the authors for their work.**

Sincerely yours,

Dr. Farah Deeba

Research Fellow

Department of Psychology

Macquarie University,

North Ryde, NSW

Australia

“**Sheet for Comments**”

Overall for the whole paper please see the format of writing for the journal and format your paper.

**Abstract**

1. Please write the name of the questionnaires sequentially as per your variables addressed in the study.
2. The long descriptions on statistical analyses run for the study is not necessary in abstract rather some values with the main findings are appreciable.
3. The information on ethics needs a reference number.

**Introduction**

The introduction is written well and concise.

**Methods**

1. Ethics information is needed to be included in the methodology section.
2. What demographic variables have you collected?? It is needed to have description on the demographic variables. For instance, different demographic information on the students, like which year, subjects, etc.
3. It would be great if you give a consort flow chart on the inclusion of the participants.
4. Please write about the questionnaires used in your study in the way the journal asks. For instance, subscales? How many?? What is Likert of 1?? What is CA (Chronbach alpha)?? Please state Chronbach Alpha for all the scales.
5. In the statistical analyses section it is needed to be described clearly about the how the multi-collinearity of the variables were assessed and how they were managed. How the correlated variables were managed in analyses for the regression.

**Result:**

1. Descriptive statistics are needed for the Demographic variables in relation to the independent variables in Table 2 (minimum and maximum are not needed)
2. Some of the descriptions are not clearly stated, like, what do you wanted to mean by money spent for FB? Is it money spent for buying data for FB or else?
3. The demographic information given in Table 1 will be better fitted in sample / participant section.
4. The description of the regression analysis is repeated on the result section and conclusion as well.

**Conclusions**

1. It is needed to be written without repeating the results in the conclusions again.

**Reference**

1. Many of the references are not in correct format, for instance they are in all capital letters’ in references
