# Supplementary material for: Facebook addiction and affected academic performance among Ethiopian university students: A cross-sectional study
Source: PLoS One. 2023 Feb 6;18(2):e0280306. doi: 10.1371/journal.pone.0280306 (PMC9901753; doi:10.1371/journal.pone.0280306)
Supplement: Attachment — Submitted filename: Response to Reviewers.docx [file pone.0280306.s004.docx]

**Response to Reviewers**

Thanks to all reviewers, they all had reported as all previously raised comments has addressed, so we didn’t get any comments at this stage except the editors’ concern of the English language, grammar usage and overall readability of the manuscript and suggested edition. Accordingly, our manuscript has copy edited for English language by professional English language academic instructor and the details of this professional has attached separately. Concerning the other suggestions like changes to financial disclosure and the references, no change has made to financial disclosure and the references were reviewed thoroughly to fulfill the journal requirement and no retracted article has been referenced.

Thank you again and we will wait for your positive response!

**Best Regards!**
